# Repeatability of Apparent Diffusion Coefficient at 3.0 Tesla in Normal Pancreas

**DOI:** 10.7759/cureus.15734

**Published:** 2021-06-18

**Authors:** Shiyue Chen, Ri Liu, Chao Ma, Yun Bian, Jing Li, Panpan Yang, Minjie Wang, Jianping Lu

**Affiliations:** 1 Radiology, Changhai Hospital of Shanghai, Shanghai, CHN

**Keywords:** magnetic resonance imaging, pancreas, dwi, adc, repeatability

## Abstract

Purpose: To evaluate the apparent diffusion coefficient (ADC) test-retest repeatability of the normal pancreas based on diffusion-weighted imaging (DWI).

Methods: Twenty-six healthy volunteers (mean 47.6 years; 13 men) were included and scanned twice with reposition for a DWI sequence at 3.0-T. Two readers measured the ADCs of pancreatic head, body and tail for two DWIs, independently. The mean ADCs of the pancreatic head, body and tail were calculated as the global pancreatic ADC. Test-retest repeatability and agreement of ADC measurement were evaluated by the Bland-Altman analysis, intra-class correlation coefficient (ICC) and coefficient of variation (CV).

Results: The global pancreatic ADC showed the best test-retest repeatability (mean difference ± limits of agreement were 0.05 ± 0.25×10^-3^ mm^2^/s; ICC, 0.79; CV, 6%). Test-retest repeatabilities for ADC of pancreatic head, body or tail were scattered, with mean difference ± limits of agreement between two tests were 0.03 ± 0.47, 0.05 ± 0.42 and 0.06 ± 0.31 (×10^-3^ mm^2^/s) (ICCs, 0.81, 0.52 and 0.68; CVs, 9%, 8% and 8%), respectively. Both intra-observer repeatability and inter-observer reproducibility were acceptable for global pancreatic ADC between measurements of the two DWIs.

Conclusions:The best test-retest repeatability of ADC in the normal pancreas was only for the whole pancreas with a CV of 6%. Cautions should be taken in interpreting longitudinal clinical changes in ADC values of the normal pancreas for the measurements do have an inherent variability by locations.

## Introduction

Magnetic resonance imaging (MRI) is an important tool for the diagnosis and evaluation of various abdominal diseases with quantitative and qualitative methods. For the imaging of the pancreas, MRI is widely used to detect and differentiate pancreatic diseases [1]. Specifically, as a quantitative MRI technology, diffusion-weighted imaging (DWI) with derived apparent diffusion coefficient (ADC) was introduced to quantify water diffusion in vivo [2]. With a quasi-exponential growth of research applications as well as clinical practice, DWI provides additional information on the pancreas as a supplement to conventional MRI techniques such as T1/T2-weighted imaging [3].

As a biomarker, ADC is helpful in the characterization of pancreatic diseases including chronic pancreatitis, cystic and solid pancreatic tumors [2]. Pancreatic cancer had lower ADC than the normal pancreas [4-6]. Ideally, changes in ADC should principally reflect the composition or cellularity of tissue, while at the same time not being susceptible to variations associated with measurement repeatability. Therefore, in order to detect a meaningful difference in ADC, it is desirable that the uncertainty of the ADC measurement should be lower than the difference between the normal and abnormal pancreatic tissues.

It is actually an important point to assess ADC repeatability in body DWI [2]. Data onto evaluating the repeatability of ADC for normal pancreas are limited: one study only reported the mean coefficient of variations (CVs) of 10.6% for ADC in the normal pancreatic body at 3.0-T [7]. As most of the studies have used the normal pancreas as a control group when comparing ADC in different pancreatic entities, the ADC test-retest repeatability in the normal pancreas was few reported, especially for different anatomical regions of the pancreas. To better understand the magnitude of a change of ADC and obtain useful metrics for ADC, the aim of this study was to investigate the ADC test-retest repeatability in the normal pancreas.

## Materials and methods

Clinical study

This prospective study was approved by the ethics review board. Before MRI examination, informed consents were signed by the volunteers. Twenty-six volunteers (mean age 47.6±10.5 years; range 24-67 years; 13 men, 13 women) with the body mass indexes (BMIs) between 18.5 kg/m^2^ and 28 kg/m^2^ were enrolled in the study at January 6 and 7, 2018. Exclusion criteria were set as subjects with pancreatic disease, diabetes, hepatic cirrhosis history and any contraindication of MRI examination.

MRI

Volunteers underwent MRI examinations at 3.0-T (MR 750, GE Healthcare, Milwaukee, USA) with a 32-element coil used for signal reception. MRI images were acquired with pancreas MRI protocol and two fat saturated axial free breathing DWIs (b-values=50 and 800 s/mm^2^). A single shot echo-planar-imaging sequence used for DWI weighted with three orthogonal gradient directions. The first DWI was acquired after the positioning of the examination and the second one was acquired with repositioning of the volunteers with a new localizer after getting the participant out and back in. Six hours fasting were required before MRI examination for all the volunteers. A median delay between two DWIs was about 10 minutes. The main parameters of DWI were: repetition time/echo time (TR/TE), 4,000/76 ms; matrix size, 132×128; field of view (FOV), 380×304 mm^2^; number of excitations (NEX), 1 and 4 for b50 and b800, respectively; slice thickness/gap, 6.0/1.0 mm; number of slices, 26; acceleration factor, 2.0; bandwidth, 250 kHz.

Data analysis

Two radiologists issued the diagnostic reports on MRI examination for all volunteer and did not find any abnormalities of the pancreas. The head, body and tail regions of the pancreas were clearly displayed on the DW images in all subjects [8].

To evaluate the variability, two observers (both with over 8 years experience in radiology) measured the ADCs of pancreatic head, body and tail for the two DWIs. One of the observers (as observer 1) measured ADCs again with an interval of four weeks for the two DWIs. Mean ADC values were obtained from an oval/round region of interest (ROI) on the ADC maps. The area of ROI was between 31 and 119 mm^2^ (mean 71.6 mm^2^). Vessels, ducts and common bile duct were avoided with reference to T2WI/T1WI in the measurements of ADC. The mean ADC values of the pancreatic head, body and tail was calculated as the global pancreatic ADC.

Statistical analysis

Statistical analyses were performed using MedCalc, version 13.0.0.0 (MedCalc Software, Ostend, Belgium). Intra-/inter-observer variability and test-retest repeatability of ADC measurements for each anatomical region of the pancreas and whole pancreas were analyzed by Bland-Altman analysis, CV and intra-class correlation coefficient (ICC: 0-0.20 = poor correlation, etc.). The first ADC measurements of observer 1 were used for the calculation of inter-observer variability. The mean ADC value of the two measurements of observer 1 was used for the further analyses. The CVs were calculated by the standard deviation and mean ADC values of the two DWIs (test and retest DWIs) for pancreatic head, body, tail and whole pancreas, respectively. ADC values between test-retest DWIs were compared by paired sample t-test. Comparisons of the mean ADCs among three anatomical regions of the pancreas were performed by using one way analysis of variance (ANOVA) and post-hoc analyses.

## Results

**Figure 1:**
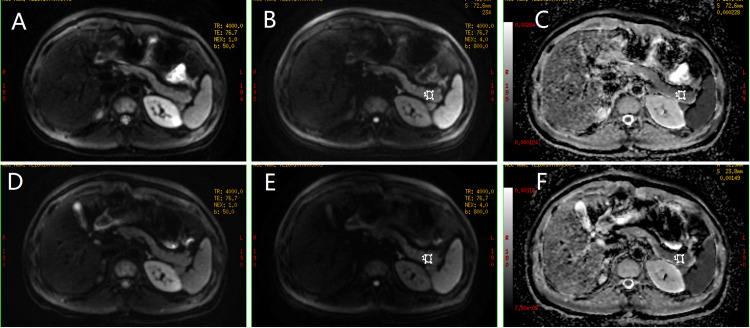
Representative images were acquired from a volunteer with test-retest DWIs. DWI images with b-value of 50/800 s/mm^2^ (A, B) and corresponding ADC map of first DWI (C); (D-F) images and the ADC map of the retest DWI. DWI - diffusion-weighted imaging ADC - apparent diffusion coefficient

The typical images of test-retest DWIs and ADC measurements were demonstrated in Figure 1.

Intra-observer variability of ADC

**Figure 2:**
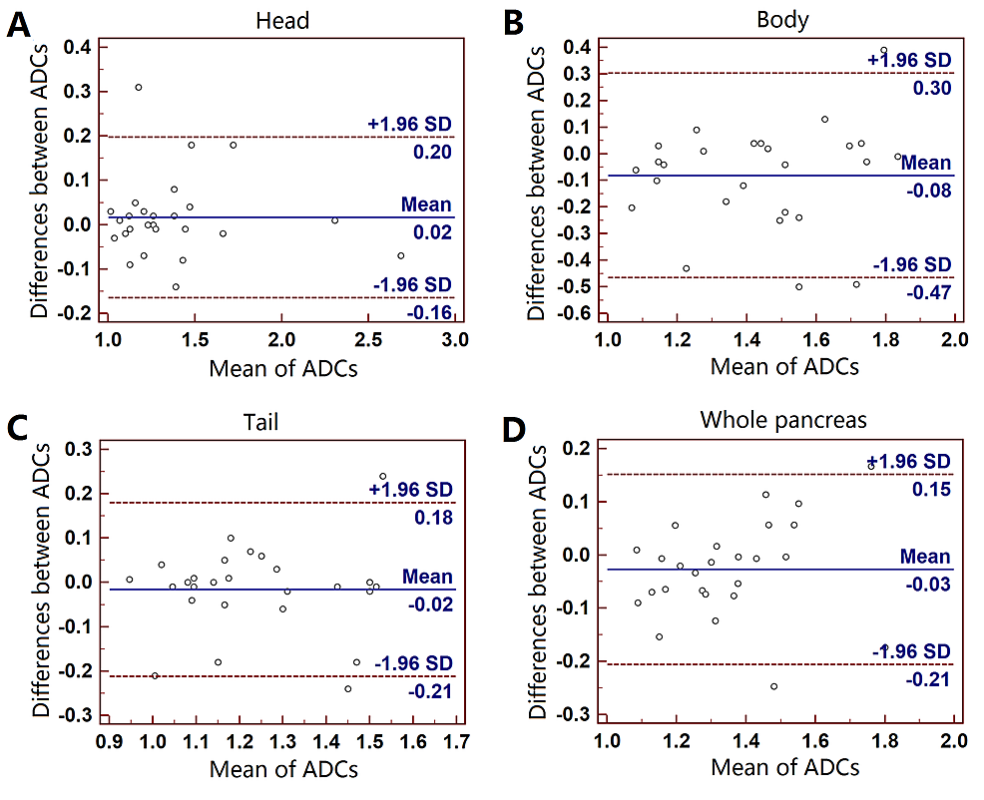
Bland-Altman plots of the intra-observer ADC measurements. Pancreatic head (A), body (B), tail (C) and whole pancreas (D) on the first DWI. Y-axis: bias of ADC measurements (×10^-3^ mm^2^/s); x-axis: the mean ADCs.

For the first DWI, the bias and limits of agreement (LOAs) between two ADC measurements were 0.02 [-0.16-0.20] ×10^-3^ mm^2^/s for pancreatic head (ICC, 0.97), -0.08 [-0.47 - 0.30] ×10^-3^ mm^2^/s for pancreatic body (ICCs, 0.71), 0.02 [-0.21 - 0.18] ×10^-3^ mm^2^/s for pancreatic tail (ICC, 0.85) and -0.03 [-0.21 - 0.15] ×10^-3^ mm^2^/s for the whole pancreas (ICC, 0.89). The mean ADCs in pancreatic body were more scattered than the other three groups. The mean CVs for the twice ADC measurements were between 3% and 7% for the four groups. ANOVA results showed that the mean ADCs of different anatomical regions were different (P < 0.05). Post-hoc analyses results demonstrated ADC was higher in pancreatic body than that in pancreatic tail (P = 0.035). Graphic illustrations of Bland-Altman analyses were shown in Figure 2. For the reposition DWI, similar results were also found as the first DWI.

Inter-observer variability of ADC

**Figure 3:**
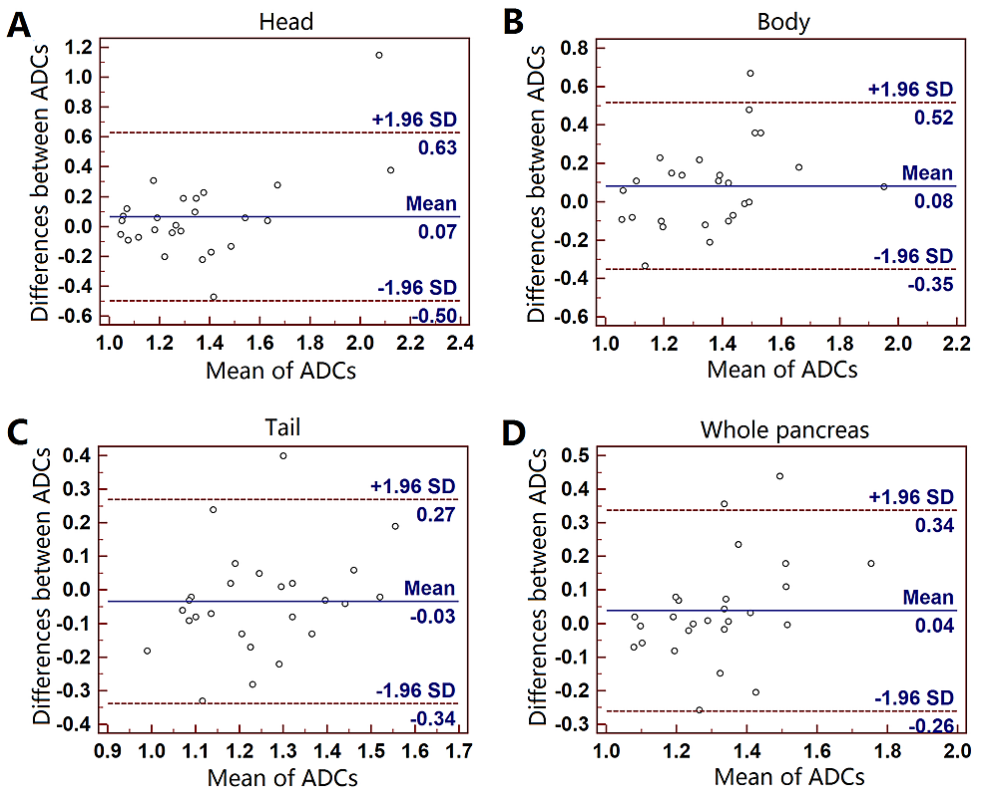
Bland-Altman plots of the inter-observer ADC measurements. Pancreatic head (A), body (B), tail (C) and whole pancreas (D) on the first DWI. Y-axis: bias of ADC measurements (×10^-3^ mm^2^/s); x-axis: the mean ADCs.

For the first DWI, the bias and LOAs between ADC measurements of two observers were 0.07 [-0.50 - 0.63] ×10^-3^ mm^2^/s for pancreatic head (ICC, 0.58), -0.08 [-0.35 - 0.52] ×10^-3^ mm^2^/s for pancreatic body (ICCs, 0.55), 0.03 [-0.34 - 0.27] ×10^-3^ mm^2^/s for pancreatic tail (ICC, 0.57) and -0.04 [-0.26 - 0.34] ×10^-3^ mm^2^/s for the whole pancreas (ICC, 0.63). The ADC of whole pancreas had the best reproducibility among the four groups. The mean CVs for the ADC measurements of two observers were between 5% and 9% for the four groups. Graphic illustrations of Bland-Altman analyses were shown in Figure 3. Similar results were also found in the reposition DWI.

Test-retest repeatability

**Figure 4:**
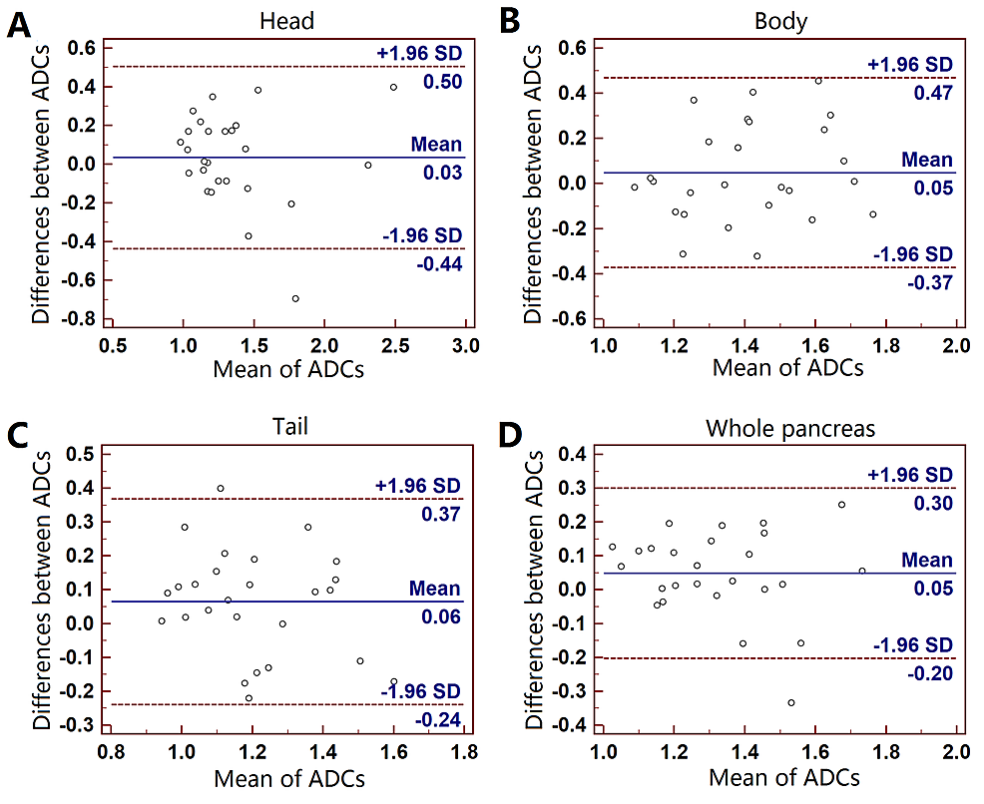
Bland-Altman plots of the mean ADC measurements on test-retest DWIs. Pancreatic head (A), body (B), tail (C) and whole pancreas (D). Y-axis: bias of ADC measurements (×10^-3^ mm^2^/s); x-axis: the mean ADCs.

For the test-retest DWIs, the mean ADC values of different regions and the whole pancreas for the two DWIs and statistical analyses results were summarized in Table 1. The mean ADC of whole pancreas had the best repeatability among the four groups. The mean ADC of pancreatic tail was lower in the reposition DWI (P = 0.045). There were no significant differences in ADCs between two DWIs at head, body or whole pancreas. Figure 4 showed the Bland-Altman analyses results.

**Table 1:** ADC values of three pancreas regions and whole pancreas for the test-retest DWIs and statistical analyses results. Data are expressed as mean ± standard deviation (×10^-3^mm^2^/s); ICC, intra-class correlation coefficient; B_A, Bland Altman; CV, coefficient of variation; ADC, apparent diffusion coefficient; ROI, region of interest. *No significant differences between ROI sizes in three regions and the whole pancreas for test-retest DWIs (P = 0.20-0.58).

	Head	Body	Tail	Whole pancreas
ADC of first DWI	1.37±0.38 (1.02-2.69)	1.43±0.24 (1.07-1.84)	1.24±0.18 (0.95-1.53)	1.35±0.19 (1.09-1.80)
ROI size (mm^2^) of first DWI*	83.2±9.0 (44-101)	75.5±15.1 (44-106)	76.6±13.4 (35-119)	235.4±30.7 (166-272)
ADC of second DWI	1.34±0.40 (0.92-2.31)	1.39±0.20 (1.07-1.83)	1.17±0.21 (0.87-1.69)	1.30±0.21 (0.96-1.70)
ROI size (mm^2^) of second DWI	84.3±7.3 (55-99)	73.7±14.3 (35-101)	73.5±11.8 (44-119)	231.5±24.8 (174-277.5)
ICC	0.81	0.52	0.68	0.79
B_A	0.03 [-0.44-0.50]	0.05 [-0.37-0.47]	0.06 [-0.24-0.37]	0.05 [-0.20-0.30]
CV	9%	8%	8%	6%
P-values	0.48	0.48	0.045	0.07

## Discussion

The current study demonstrated that ADCs of the normal pancreas and test-retest repeatability were dependent on the different anatomical regions of the pancreas. Only the repeatability of the mean ADC of the whole pancreas was acceptable because the test-retest bias of ADC measurements less than ±0.10×10^-3^ mm^2^/s and the LOAs were less than ± 0.30×10^-3^ mm^2^/s [9]. The standardization of ADC measurement methods of the pancreas is important to the healthy subjects as the control group in studies. The results of the current study recommended that caution should be taken in interpreting longitudinal clinical changes in ADC values of the pancreas for the measurements do have an inherent variability by location.

As a biomarker, the evaluation of repeatability in ADC measurements is important to diagnose diseases or to detect a meaningful change in treatment. ADC repeatability has already been evaluated over time for breast [10], lung [11,12] and liver [13,14], but only a few studies have evaluated the test-retest ADC repeatability in the pancreas. Rosenkrantz et al. found that test-retest ADC repeatability was moderate in the normal pancreatic body both at 1.5-T and 3.0-T [7]. However, the test-retest ADC repeatability was not acceptable to three anatomical regions of the pancreas in the study. The inconsistent findings between the two studies may be caused by the differences of the parameters in DWI, specifically in b-values. In this study, two b-values were used for DWI, and the choosing of the b-values is according to the recommendation of the ISMRM-sponsored workshop [15]. The other two studies reported that there was no significant effect of MRI systems on ADCs reproducibility of the normal pancreas (including head, body and tail) over a short-term of the normal pancreas at 1.5-T and 3.0-T for a given individual, respectively [16,17]. Barral et al. investigated the reproducibility and variations in ADCs of the normal pancreas using repeated measurements method both at 1.5- and 3.0-T and found that intra- and inter-observer ADC measurements were acceptable and ADCs in different segments of the pancreas were homogeneous at 3.0-Tesla [18]. In the current study, we also evaluated the intra-/inter-observer variability in ADC measurements of three regions of the normal pancreas at 3.0-T, as well as the whole pancreas on ADC measurements for test-retest DWIs. However, the intra-observer repeatability of mean ADCs in the pancreatic body was not acceptable. We found that the ADC values were the lowest in the pancreatic tail, which was similar as being showed in some studies [19,20]. In addition to the pancreas composition, DWI acquisition parameters, including field strength, b-values selection, respiratory compensation acquisition, and post-processing approach may influence the ADCs of the pancreas [21]. The inconsistent findings in the study might be the differences in b-values and field strength. Additionally, the free-breathing technique used in the study might have some effects on the findings. The finding of a significantly lower ADC in the pancreatic tail in the second ADC measurement compared to the first one may be due to the effects of these factors. To limit the possible influence of field heterogeneity, b-values and acquisition methods on the ADC measurements, a normalized ADC method is a promising tool [22-24]. An average value of three delineations will be more robust than the three small delineations. One of the main reasons why we investigated the global pancreatic ADC value is to consider the possible effect of the gradient nonlinearity (GNL) on the pancreatic ADC because the pancreas is about 15 cm long in the abdominal cavity behind the stomach. Previous multi-scanner quality control studies have demonstrated that significant ADC errors (ranging from +25% to -50%) were detected away from the magnet isocenter for many MRI systems [25-27].

The current study had some limitations. First, all MRI exams were performed on a 3.0-T scanner within two days, this ideal experimental scheme might be difficult to achieve in daily work. Second, as GNL of the MRI system causes the spatial nonuniformity to ADCs and this fact may have an impact on the findings [27]. Third, relatively low b-values (50 and 800 s/mm^2^) was used to reduce motion artifacts and to keep a good signal-to-noise ratio in abdominal DWI, but DWI with higher b-values might improve the pancreatic tumor delineation [28]. Furthermore, only two b-values were used in the study to reduce examination time, although multiple b-values DWI might be more accurate to the calculation of ADCs. Finally, the effects of field strength and different b-value selection on ADC measurements of the pancreas were not evaluated, which may affect the results.

## Conclusions

This study demonstrated that the ADC measurements of the pancreas are technically challenging and repeatability is variable. The best test-retest repeatability of ADC in normal pancreas was only for the whole pancreas with CV of 6%. Cautions should be taken in interpreting longitudinal clinical changes in ADC values of the normal pancreas for the measurements do have an inherent variability by location.
